# Cable Tension Monitoring Based on the Elasto-Magnetic Effect and the Self-Induction Phenomenon

**DOI:** 10.3390/ma12142230

**Published:** 2019-07-10

**Authors:** Senhua Zhang, Jianting Zhou, Yi Zhou, Hong Zhang, Jingwen Chen

**Affiliations:** 1 College of Civil Engineering, Chongqing Jiaotong University, Chongqing 400074, China; 2 Chongqing Yapai Bridge Engineering Quality Inspection Co., Ltd., Chongqing 401120, China

**Keywords:** steel cable, cable-supported structure, tension monitoring, self-induction, elasto-magnetic induction

## Abstract

Cable tension monitoring is important to control the structural performance variation of cable-supported structures. Based on the elasto-magnetic effect and the self-induction phenomenon, a new non-destructive evaluation method was proposed for cable tension monitoring. The method was called the elasto-magnetic induction (EMI) method. By analyzing the working mechanism of the EMI method, a set of cable tension monitoring systems was presented. The primary coil and the induction unit of the traditional elasto-magnetic (EM) sensor were simplified into a self-induction coil. A numerical analysis was conducted to prove the validity of the EMI method. Experimental verification of the steel cable specimens was conducted to validate the feasibility of the EMI method. To process the tension monitoring, data processing and tension calculation methods were proposed. The results of the experimental verification indicated that different cables of the same batch can be calibrated by one proper equation. The results of the numerical analysis and the experimental verification demonstrated that the cable tension can be monitored both at the tension-applying stage and the tension-loss stage. The proposed EMI method and the given monitoring system are feasible to monitor the cable tension with high sensitivity, fast response, and easy installation.

## 1. Introduction

Cable-supported structures are extensively applied in infrastructure construction, such as in bridges and roofs [1]. Steel cables are the main bearing members of the cable-supported structures group. The cables ensure the bearing capacity of the cable-supported structures [2]. The tension of the cable starts changing since the steel cable has been installed and tensed. Cable tension varies because of various factors such as the performance of the materials, the construction situation, and the external environment [3,4]. Variation of the cable tension often leads to structural performance variation of cable-supported structures [5,6]. Therefore, the cable tension should be monitored accurately.

In view of this necessity, cable tension monitoring has been studied by many researchers who work on non-destructive evaluation (NDE) and structural health monitoring [7,8,9,10,11,12]. However, the actual tension monitoring for in-service cable-supported structures remains a tough task [13]. The traditional tension monitoring method using a strain gauge can only measure the relative variation of the cable tension (not the actual tension). The strain gauge’s deformation and the structure’s deformation need to be transmitted by a coupling agent [14,15]. This generates a lag in the deformation of the strain gauge, which makes this method unsuitable for real-time measurement. The vibrating frequency method is based on the cable tension and the vibration modal parameters of the cable [2,16]. The accuracy of the tension detection is not high [17], because the vibrating frequency method is affected by many factors. These factors include the location of the sensor, the polyethylene (PE) jacket of the cable, the span of the cable, the sag of the cable, the slant of the cable, the boundary conditions of the cable, and the correctness of the calculation model [18]. The acoustoelastic effect method obtains cable tension by measuring the propagation velocity of the ultrasonic wave within the cable [19,20,21]. Due to the complexity of the cables, establishing suitable dispersion equations is hard. The common strategy is to simplify cables into steel rods to calculate the dispersion curves under different tension levels. Then, the relationship between the propagation velocity of the ultrasonic guided wave and the cable tension can be established. Besides, the acoustoelastic effect method is often used to measure the average tension within a length of the cable. When the measured cable contains a defect, there will be an error in the test result. The eddy current method is a non-destructive testing method. The eddy current method is based on the principle of electromagnetic induction [22,23]. The detection of the cable tension is achieved by monitoring the change of the coil’s impedance, which is caused by the eddy current field in conductive materials. This method has the advantages of high sensitivity, no close contact with the tested object, and no coupling agent [24]. However, the eddy current method cannot avoid the skin effect [25]. The eddy current method is unsuitable for monitoring cables whose diameters are large.

The method based on the elasto-magnetic (EM) effect is promising to monitor the stress of ferromagnetic materials [26,27,28]. This method overcomes most of the disadvantages encountered in the above methods [29]. According to the EM effect, an excitation magnetic field is required to magnetize the ferromagnetic component. The ferromagnetic component’s stress can be ascertained by measuring the change of the magnetic permeability of the component [30,31]. The existing EM-based sensor contains a primary coil and an induction unit [32]. Therefore, the existing EM-based sensors are hard to install [26,29,33,34]. These bottlenecks are calling for a feasible method which can monitor the cable tension in a real-time, accurate, and simple way.

The magnetic properties of ferromagnetic materials depend on their stress state. More importantly, the magnetic properties also relate to their stress history. Therefore, the magnetic properties of steel cables are different at the tension-applying stage and at the tension-loss stage [35,36]. A lot of researches have been done on cable tension monitoring, but most of the researches were focused on the tension-applying stage. In most of the researches, the maximum cable tensions were no more than 40 kN [33,37,38,39]. This maximum cable tension was different from the cable tensions used in actual engineering. Thus, studying the tension monitoring of the cables under a high tension level is necessary. Studying the tension monitoring of the cables at the tension-applying stage and at the tension-loss stage is also necessary.

In this paper, an NDE method was proposed for monitoring the cable tension. The proposed method was based on the EM effect and the self-induction phenomenon. This method was called the elasto-magnetic induction (EMI) method. The primary coil and the induction unit of the traditional EM sensor were simplified into a self-induction coil. The working mechanism of the EMI method was analyzed. A set of cable tension monitoring systems was presented. Using the finite element analysis software COMSOL, a numerical analysis was conducted to prove the validity of the EMI method. Cable tension monitoring experiments were conducted. The aim of the experiments was to validate the EMI method’s feasibility. The experiments were conducted both at the tension-applying stage and at the tension-loss stage. The results of the numerical analysis and the experiments were consistent. The results demonstrated that the EMI method and the given system are feasible to monitor cable tension with high sensitivity, fast response, and easy installation, apart from the advantages of the traditional EM sensor.

## 2. Working Mechanism

According to the EMI method, the self-induction coil can act as the primary coil and the induction unit. Driven by an excitation current, the self-induction coil provides an excitation magnetic field for cables. The excitation magnetic field can be expressed as Equation (1), where H is the intensity of the excitation magnetic field, N is the number of turns of the self-induction coil, I is the intensity of the excitation current, and l_m_ is the length of the self-induction coil.

(1) $$H\;=\;\frac{\mathrm{NI}}{l_{m}}$$

Magnetization intensity is the representative magnetic property of the steel cable. The magnetization intensity changes with the stress of the cable [40,41]. According to the famous Joule theory, the EM effect can be described as Equation (2) [38]. In Equation (2), ε is the cable’s axial strain, λ_s_ is the cable’s axial deformation constant, M_s_ is the cable’s saturation magnetization, K_u_ is the cable’s uniaxial magnetic anisotropy constant, θ_0_ is the cable’s angle between the direction of the excitation magnetic field and the easy-magnetized axis of the cable, σ is the cable’s stress, E is the cable’s elasticity modulus, and ∆M is the change of the cable’s magnetization intensity.

(2) $$\varepsilon \;=\;\frac{\Delta l}{l}\;=\;\frac{{3\lambda }_{s}M_{s}}{{2K}_{u}}{\Delta \mathrm{Msin}}^{2}\theta_{0}{\cos\theta }_{0}\;=\;\frac{\sigma }{E}$$

When I is a constant, according to the magnetization theory of ferromagnetic materials, the change of the cable’s magnetization intensity can be expressed as Equation (3) [42]. In Equation (3), ∆M is the change of the cable’s magnetization intensity, H is the intensity of the excitation magnetic field, μ_ic_ is the magnetic permeability of the cable, and μ_ic0_ is the cable’s magnetic permeability at zero stress.

(3) $$\Delta M\;=\;H\times \left(\mu_{\mathrm{ic}}{-\mu }_{\mathrm{ic}0}\right)$$

(4) $$L\;=\;\left(\frac{\mu_{\mathrm{ic}}A_{\mathrm{ic}}{+\mu }_{\mathrm{air}}A_{\mathrm{air}}}{l_{m}}\right)N^{2}$$

The self-induction phenomenon was described as Equation (4), where L is the inductance of the self-induction coil, μ_ic_ is the magnetic permeability of the cable, A_ic_ is the sectional area of the cable, and A_air_ is the sectional area of two parts: Part one is the polyvinyl chloride (PVC) skeleton of the self-induction coil, and part two is the air ring between the PVC skeleton and the cable. The PVC skeleton’s magnetic permeability and the air ring’s magnetic permeability are represented by μ_air_. In Equation (4), l_m_ is the length of the self-induction coil and N is the number of turns of the self-induction coil.

According to Faraday’s law of electromagnetic induction, the inductance of the self-induction coil is determined by the self-induction coil and the cable. Acting as an induction unit, the self-induction coil can convert the cable’s magnetic permeability into the self-induction coil’s inductance. Combining the EM effect and the self-induction phenomenon, the dependence of the cable’s stress (σ) on the self-induction coil’s inductance can be deduced from Equations (1)–(4). With a certain excitation current, the function of the cable’s stress and the self-induction coil’s inductance can be described as Equation (5). In Equation (5), E is the cable’s elasticity modulus, λ_s_ is the cable’s axial deformation constant, M_s_ is the cable’s saturation magnetization, K_u_ is the cable’s uniaxial magnetic anisotropy constant, θ_0_ is the cable’s angle between the direction of the excitation magnetic field and the easy-magnetized axis of the cable, I is the intensity of the excitation current, N is the number of turns of the self-induction coil, A_ic_ is the sectional area of the cable, L is the inductance of the self-induction coil, and L_0_ is the self-induction coil’s inductance when the cable stress is zero.

(5) $$\sigma \;=\;E\frac{{3\lambda }_{s}M_{s}}{{2K}_{u}}\sin^{2}\theta_{0}{\cos\theta }_{0}\frac{I}{N\times A_{\mathrm{ic}}}\left({L-L}_{0}\right)$$

(6) $${\sigma \;=\;K}_{\mathrm{con}}\left({L-L}_{0}\right){\;=\;K}_{\mathrm{con}}\times \Delta L$$

For the specific steel cable and the specific self-induction coil, E, λ_s_, M_s_, K_u_, θ_0_, and N were constants. Thus, Equation (5) can be described as Equation (6), where K_con_ is a constant, L is the inductance of the self-induction coil, and L_0_ is the self-induction coil’s inductance when the cable stress is zero. According to Equation (6), there existed a linear relationship between the self-induction coil’s inductance increment (∆L) and the cable’s stress, which means the cable tension can be ascertained by measuring the inductance of the self-induction coil.

**Figure 1:**
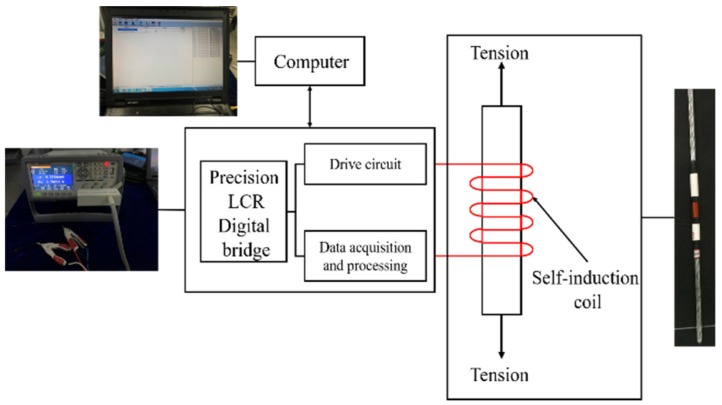
Elasto-magnetic induction (EMI) detecting system frame.

**Figure 2:**
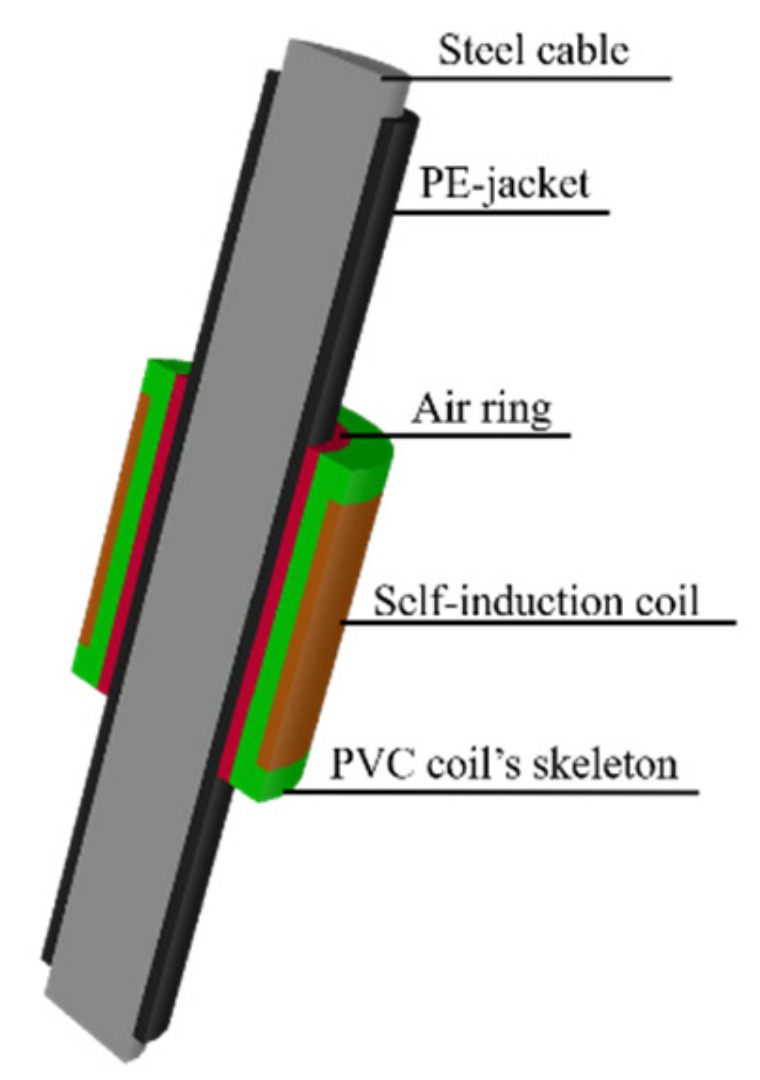
Structure of the self-induction coil.

The cable tension monitoring was realized by the EMI detecting system. As shown in Figure 1 and Figure 2, the EMI detecting system contains a precision LCR (Inductance, Resistance and Capacitance) digital bridge, a computer, a self-induction coil, and a steel cable. The precision LCR digital bridge contains a drive circuit, and a data acquisition and processing module. To generate the necessary magnetic field, the drive circuit inputs an excitation current into the self-induction coil. According to the EM effect, the change of the cable tension results in a variation of the cable’s magnetic permeability. Then, the self-induction coil converts the variation of the cable’s magnetic permeability into the easily measured inductance. Since the self-induction coil’s inductance is measured by the precision LCR digital bridge, the cable tension is calculated by the computer.

## 3. Numerical Analysis 

Using the finite element analysis software COMSOL, the finite element model was established. The numerical analysis was conducted to analyze the monitoring of the cable tension based on the EMI method. With a certain excitation current, the simulation results of different cable tensions were collected and analyzed to prove the validity of the EMI method.

**Figure 3:**
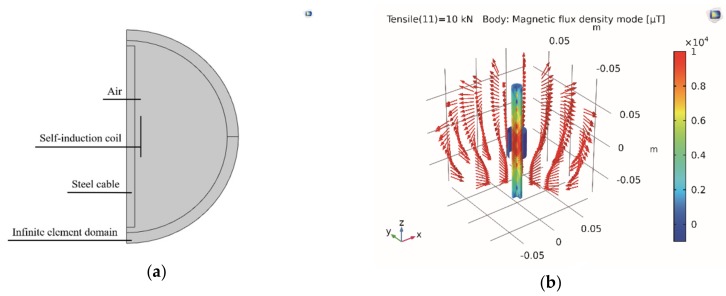
Numerical simulation process: (**a**) 2D plan model and (**b**) 3D drawing of the magnetic flux density.

The solid mechanics module and the magnetic fields module were employed to process the steady-state analysis. These two modules were coupled by the magnetostriction effect [38,43]. To simplify the computational process, the half-symmetrical 2D plan model was derived from the 3D model, as shown in Figure 3a. The infinite element method was employed to simulate the magnetic insulation boundary of the air domain. Thus, the magnetic flux could not diverge beyond the modeling domain. In addition, the cable was magnetostrictive. The saturation magnetization of the cable was 1.5 × 106 A/m. The initial magnetic susceptibility of the cable was 200. The saturation magnetostriction of the cable was 200 ppm. The magnetic field distribution around the cable was obtained by the numerical analysis. A representative step of the simulated results is shown in Figure 3b. The cable tension of the representative step was 10 kN. The self-induction coil was placed at the cable’s axial center. The maximum magnetic intensity occurred in the cable’s axial center.

**Figure 4:**
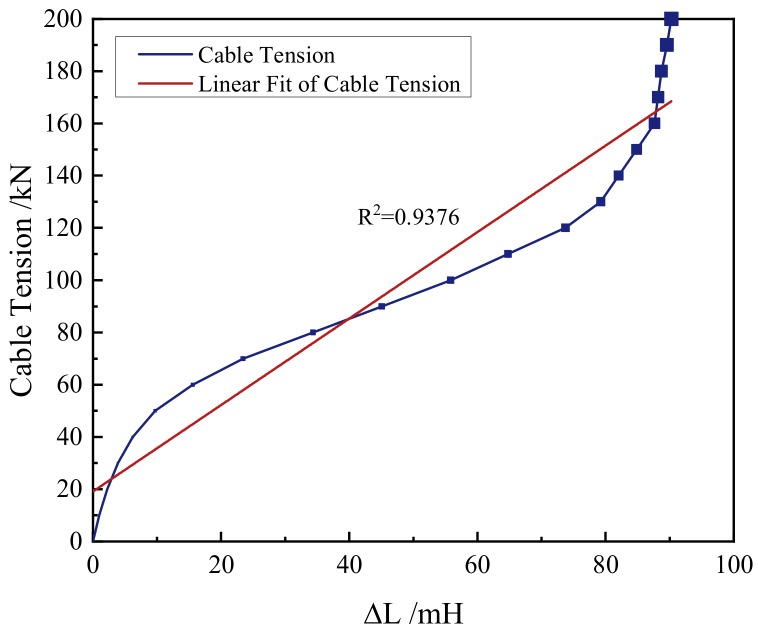
Inductance increments (∆L) of the self-induction coil obtained from the solution of the finite element model.

Collected from the results of the numerical simulation, the inductance increments of the self-induction coil are shown in Figure 4. The cable tension varied with the self-induction coil’s inductance increment in approximate linearity. As shown in Figure 4, the inductance increment increased with the increase of the tension. Taking the inductance increment as the independent variable and the tension as the dependent variable, it can be found that the tension increased with the increase of the inductance increment. The goodness of the linear fit (R^2^) was 0.9376. This demonstrated that the cable tension can be ascertained, since the inductance increment of the self-induction coil has been measured. The results of the numerical simulation proved the validity of the EMI method. In practical application, the relation curve of the cable tension and the self-induction coil’s inductance increment can be obtained by the advance calibration. An artificial intelligence algorithm such as the genetic algorithm can be employed to automatically ascertain the cable tension by the measured inductance increment of the self-induction coil [44,45]. 

## 4. Experimental Verification 

### 4.1. Materials and Methods

The cable tension monitoring experiment was carried out at room temperature. The experiment was carried out to verify the correctness of the numerical analysis’ results and the sensitivity of the EMI method. Fifteen cable specimens were tested to prove the repeatability of the experimental results. The specimens were labeled as G1-1–G1-5, G2-1–G2-5, and G3-1–G3-5. Each specimen was a single-strand wire which contained seven steel wires. Each specimen owned a PE jacket. The outer diameter of each specimen was 15.2 mm. The length of each specimen was 80 cm. To ensure the universal test machine could hold the specimen steady, the 25 cm long PE jackets were removed at each end of the specimen. The mechanical parameters of the cable specimens are presented in Table 1. 

Three self-induction coils were prepared to conduct the experiment. Each self-induction coil was winded on the PVC skeleton. The outer diameter of the PVC skeleton was 25 mm. Each self-induction coil was winded 300 times by the 0.35 mm (diameter) coated encased copper wires. The numbers of layers of the self-induction coils were set differently. The aim of the setting was to figure out the influences of the self-induction coil’s length on the accuracy and sensitivity of the cable tension monitoring. The self-induction coils were labeled as G1, G2, and G3. The parameters of the self-induction coils are presented in Table 2. To mount the self-induction coil at the middle of the specimen, sponges were used to fill the air ring between the PVC skeleton and the specimen.

**Figure 5:**
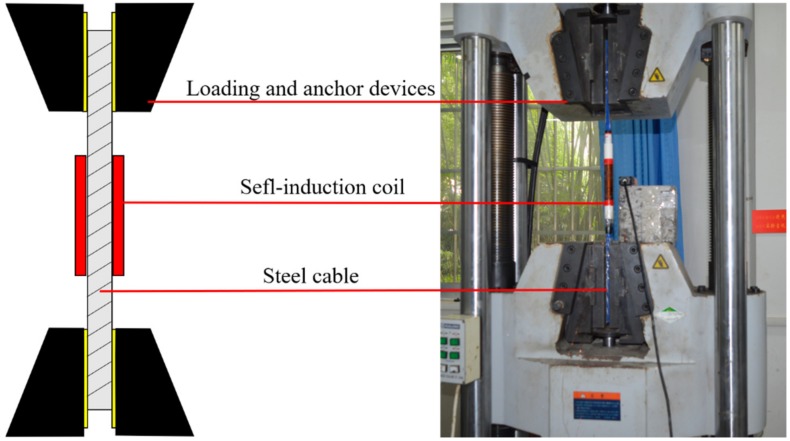
Diagram of the experimental setup.

In the experiment, a precision LCR digital bridge was employed to provide the excitation current and measure the self-induction coil’s inductance. The precision LCR digital bridge was the TH2832 Series of Compact LCR Meter, which was produced by the Changzhou Tonghui Electronic Co., Ltd (Changzhou, China). The series model was used to process the measurement. The internal resistance of the precision LCR digital bridge was set at 30 Ω. The excitation current was a sinusoidal AC signal with a 20 Hz exciting frequency. The excitation current was 66.67 mA. The universal test machine was used to stretch the specimens to each tension level, as shown in Figure 5. The universal test machine was from the E45.205 series, and it was produced by MTS Industrial Systems (Shanghai, China) Co., Ltd. The max load of the universal test machine was 100 t.

The experiment was conducted both at the tension-applying stage and at the tension-loss stage. At the tension-applying stage, the tension increased from 0 to 200 kN, with a step of 10 kN. At the tension-loss stage, the tension decreased from 200 to 0 kN, with a step of 10 kN. Both the speed of the tension application and the speed of the tension loss were 0.5 kN/s. Under each tension level, the tension was maintained for 30 s to conduct the measurement. The measurement was conducted 10 times under each tension level. The measurement was done within the first 25 s of the tension maintenance. Therefore, stable magnetization could be realized and precise data could be collected. In addition, the data collected under each tension level were averaged automatically.

### 4.2. Experimental Results

**Figure 6:**
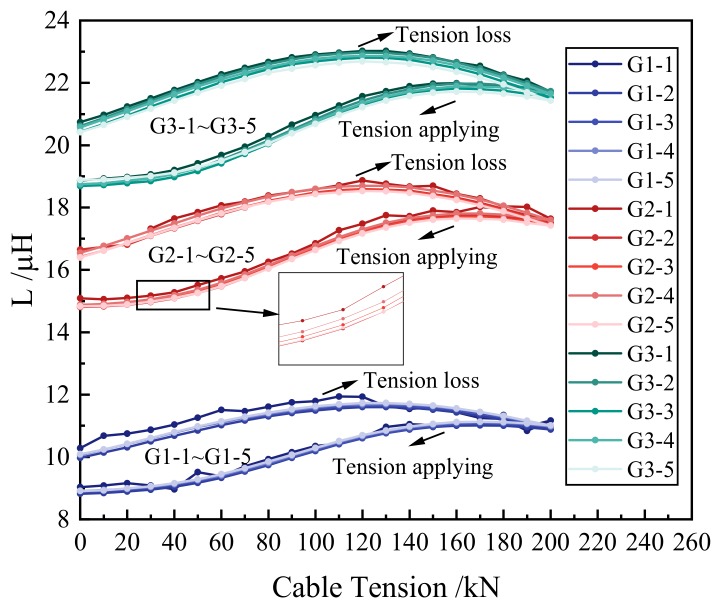
Inductances (L) under different tension levels of each specimen.

The experimental results were analyzed to validate the relation between the inductance of the self-induction coil and the cable tension. As shown in Figure 6, the inductances of the self-induction coils were obtained under different tension levels. The curves of the tension-applying stage and the curves of the tension-loss stage were different. At the tension-applying stage, the inductances increased first and then decreased with the increase of the tension. At the tension-loss stage, the inductances increased first and then decreased with the decrease of the tension. The tension of the turning point of the tension-applying stage was lower than that of the tension-loss stage. The differences were caused by the magnetic hysteresis of ferromagnetic materials [35]. The relations between the inductance and the cable tension were derived from the experimental results. Both the relation derived from the tension-applying stage and the relation derived from the tension-loss stage are discussed.

**Figure 7:**
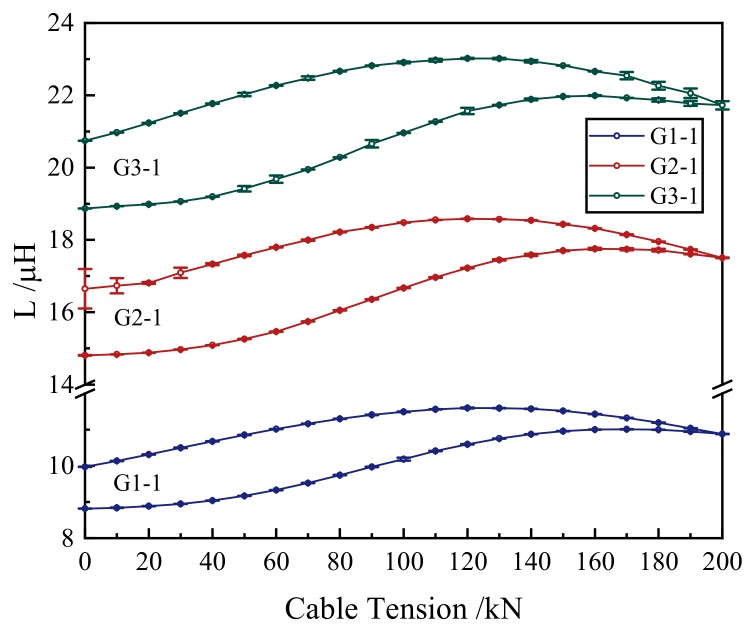
Inductances (L) under different tension levels of G1-1, G2-1, and G3-1.

The inductance–tension curves which were collected by the same self-induction coil are shown in Figure 6. These curves had good repeatability. This indicated that different cables of the same batch can be calibrated by one proper equation. Thus, there is no need to calibrate each cable. The inductance–tension curves of G1-1, G2-1, and G3-1 were added with error bars, as shown in Figure 7. Under each tension level, the inductances collected by the self-induction coils had good stability.

Under a certain tension level, the inductances collected by different self-induction coils were different. The reason was that the length of each self-induction coil was different. However, the relations between the inductances and the cable tensions were similar for different self-induction coils. This demonstrated that the length of the self-induction coil does not affect the relation between the inductance and the cable tension. For the tension-applying stage and the tension-loss stage, the sums of the absolute values of change of the inductance between each two tension levels were calculated. The absolute cumulative relative change rates of the inductance in relation to the tension were calculated, as shown in Table 3. The absolute cumulative relative change rates were calculated to analyze the sensitivity of the inductance to the cable tension.

For a self-induction coil, the measuring sensitivity of the tension-applying stage was basically the same as that of the tension-loss stage. When the number of turns of the coil was a constant, the tension monitoring sensitivity increased with the decrease of the coil’s length. This experimental result coincided with the results of the working mechanism analysis. Besides, when the number of the coil’s layers changed from two to three, the increase in the monitoring sensitivity was very small. Therefore, shortening the coil length further for higher sensitivity was unnecessary. The experimental result of specimen G3-1 is mainly discussed.

**Figure 8:**
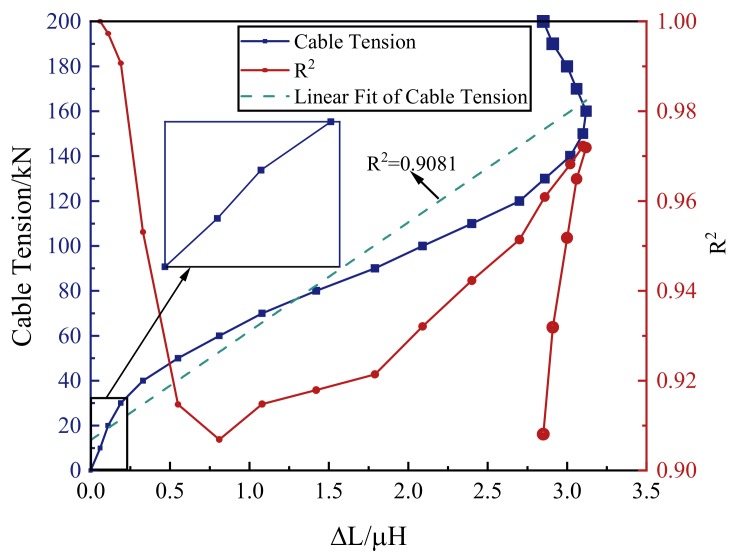
The self-induction coil’s inductance increment and the goodness of the linear fit obtained from specimen G3-1 under each tension level of the tension-applying stage.

According to Equation (6), there was a linear relationship between the inductance increment (∆L) and the cable tension. The inductance increments under different tension levels of the tension-applying stage are shown in Figure 8. At the tension-applying stage, the inductance increment increased first and then decreased with the increase of the tension. Taking the inductance increment as the independent variable and the tension as the dependent variable, it can be found that the tension increased first and then decreased with the increase of the inductance increment. The goodness of the linear fit (R^2^) was used to represent the linearity of the tension with the inductance increment under each tension level. The R^2^ of each tension level was calculated from the data in the section, from no tension to the current tension level. When the cable tension was less than 40 kN, the linearity between the cable tension and the inductance increment was good. Each R^2^ was greater than 0.99. This result coincided with Wang’s and Duan’s work [22,24]. Therefore, when the tension is small, the cable tension can be characterized precisely by the inductance of the self-induction coil.

As shown in Figure 8, the R^2^ decreased first with the increase of the tension. When the tension was greater than 50 kN, the R^2^ increased with the increase of the tension. When the tension was greater than 140 kN, the R^2^ began to decrease. Under each tension level of the tension-applying stage, the R^2^ was greater than 0.9. For the entire tension-applying stage, the cable tension varied with the inductance increment in approximate linearity, with R^2^ = 0.9081. This experimental result indicated that, at the tension-applying stage, the cable tension can be ascertained by the self-induction coil’s inductance increment (∆L).

**Figure 9:**
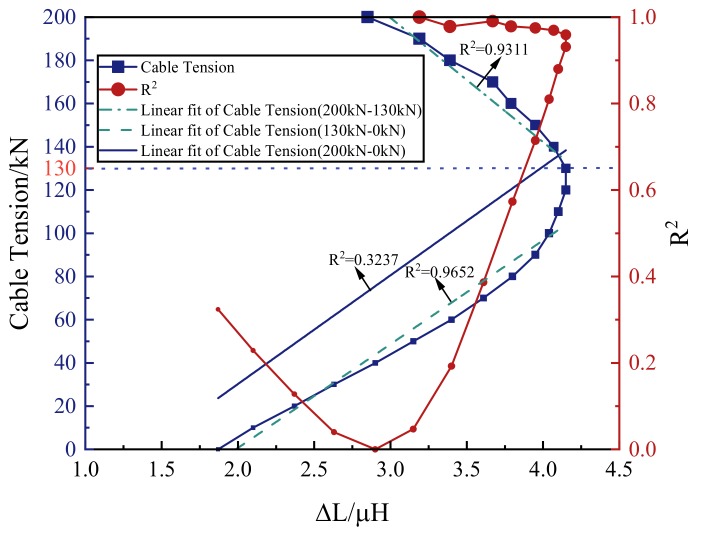
The self-induction coil’s inductance increment and the goodness of the linear fit obtained from specimen G3-1 under each tension level of the tension-loss stage.

The relation between the inductance increment and the cable tension of the tension-loss stage was different from that relation for the tension-applying stage. This difference was caused by the magnetic hysteresis of the ferromagnetic materials. The magnetic hysteresis of the ferromagnetic materials indicated that the magnetic properties of ferromagnetic materials were stress history-dependent [35]. The inductance increments of the self-induction coil under different tension levels of the tension-loss stage are shown in Figure 9. The goodness of the linear fit (R^2^) was used to represent the linearity of the tension with the inductance increment under each tension level. The R^2^ of each tension level was calculated from the data in the section from the maximum tension level (200 kN) to the current tension level. At the tension-loss stage, the inductance increment increased first and then decreased with the decrease of the cable tension. The turning point was 130 kN. Before the tension dropped below 130 kN, the R^2^ was greater than 0.9. When the tension dropped below 130 kN, the inductance increment decreased with the decrease of the tension. The inductance increment increased when the tension dropped below 40 kN. For the entire tension-loss stage, the tension cable varied with the inductance increment in poor linearity, with R^2^ = 0.3237. In addition, when the tension was greater than 40 kN, one inductance increment corresponded to two different cable tensions. Thus, at the tension-loss stage, the cable tension cannot be calculated by the linear equation of the cable tension and the inductance increment.

As shown in Figure 9, the inductance increment increased with the decrease of the tension until the tension dropped below 130 kN. When the cable tension dropped below 130 kN, the inductance increment decreased with the decrease of cable tension. Therefore, when the inductance increment was observed to drop, it can be considered that the cable tension has dropped to a low tension level (130 kN) relative to the design tension (200 kN). 

The whole tension-loss stage can be divided into two parts. The first part was from 200 to 130 kN. The second part was from 130 to 0 kN. For cable-supported structures, the losses of the cable tension were relatively small. The cable tension can be calculated with the coil’s inductance increment. The calculation was based on the linear fit results of the first part of the tension-loss stage. In this condition, the goodness of the linear fit was 0.9311. When the inductance increment began to decrease, the cable tension can also be calculated with the coil’s inductance increment. The calculation was based on the linear fit results of the second part of the tension-loss stage. In this condition, the goodness of the linear fit was 0.9652. Combining the linear fit results obtained in two parts of the tension-loss stage, the cable tension can be determined by the inductance of the self-induction coil.

**Figure 10:**
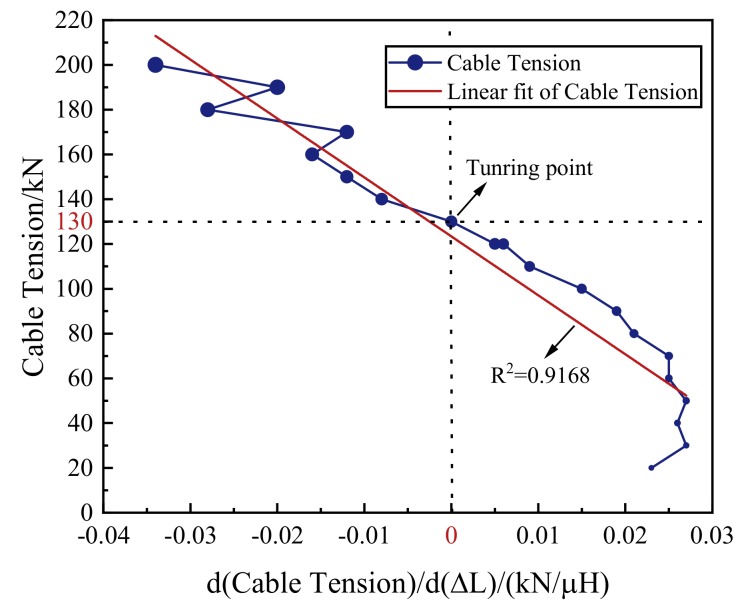
The slope of the tension–inductance increment curve and the cable tension obtained from specimen G3-1.

The inductance increment increased first and then decreased with the decrease of the tension, as shown in Figure 9. The slope of the tension–inductance increment curve kept increasing, as shown in Figure 10. The cable tension can be uniquely determined by the slope. There is a linear relationship between the cable tension and the slope of the curve, with the goodness of the linear fit R^2^ = 0.9168. The linear regression results of the cable tension agreed well with the actual cable tension. This demonstrated that the cable tension can be determined by the self-induction coil’s inductance. The experimental results verified that the given monitoring system based on the EMI method can be applied to monitor the cable tension both at the tension-applying stage and at the tension-loss stage.

Alternatively, the relation curve of the cable tension and the self-induction coil’s inductance can be obtained by the advance calibration. The cable tension can be represented by the inductance of the self-induction coil, even if the steel cable’s design tension is high or the tension monitoring requires high accuracy. 

The data of each specimen were collected by different self-induction coils. The data were processed by the above calculation methods. At the tension-applying stage, the cable tension was calculated by the linear equation of the cable tension and the inductance increment. At the tension-loss stage, the cable tension was calculated by the linear equation of the cable tension and the slope of the tension–inductance increment curve. The first aim of the processing was to figure out the influence of the self-induction coil’s length on the accuracy of the tension monitoring. The second aim of the processing was to prove the reliability and repeatability of the EMI method. The goodness of the linear fit of each specimen is shown in Table 4.

As shown in Table 4, the average R^2^ of each self-induction coil was greater than 0.9. The differences in each self-induction coil’s average R^2^ were small. The results demonstrated that the length of the self-induction coil has little effect on the accuracy of the cable tension monitoring. In addition, this result indicated that the cable tension can be monitored by the EMI method with good reliability and repeatability.

## 5. Conclusions

This paper proposed an NDE method, called the EMI method, based on the EM effect and the self-induction phenomenon. The main conclusions can be drawn:

(1) The traditional EM sensor’s primary coil and induction unit were simplified into a self-induction coil. By analyzing the EMI method’s working mechanism, a set of cable tension monitoring systems was presented. The EMI method’s correctness was proved by the numerical analysis. The experiments were carried out to verify the results of the numerical analysis.
(2) Based on the experimental results, the monitoring data processing and tension calculation methods were proposed. The methods were suitable for the tension-applying stage and the tension-loss stage. The results proved that the relation between the inductance increment and the cable tension of the tension-loss stage is different from that relation of the tension-applying stage. The results indicated that different cables of the same batch can be calibrated by one proper equation. The results demonstrated that the length of the self-induction coil has little effect on the accuracy and sensitivity of the cable tension monitoring.
(3) The results of the numerical analysis and the experiments proved that the cable tension of the cable-supported structures can be monitored both at the tension-applying stage and at the tension-loss stage. The proposed EMI method and the given monitoring system are feasible to monitor the cable tension with high sensitivity, fast response, and easy installation, apart from the advantages of the traditional EM sensor.

## Figures and Tables

**Table 1:** Mechanical parameters of the cable specimens.

Nominal Diameter/mm	Tensile Strength/MPa	Limit Load/kN	Yield Load/kN
15.2	1860	259	220

**Table 2:** Parameters of the self-induction coils.

Label	Number of Turns	Number of Layers	Length/mm	Tested Specimens
G1	300	1	105	G1-1~G1-5
G2	300	2	52.5	G2-1~G2-5
G3	300	3	35	G3-1~G3-5

**Table 3:** Absolute cumulative relative change rates of the inductance to the tension.

Specimen Label	Absolute Relative Change Rate of the Inductance to the Tension	
	Tension-Applying Stage	Tension-Loss Stage
G1-1	1.16%	1.17%
G2-1	1.60%	1.51%
G3-1	1.70%	1.79%

**Table 4:** Goodness of the linear fit (R^2^) of each specimen.

Self-Induction Coil	Specimen	R^2^ of the Tension-Applying Stage	R^2^ of the Tension-Loss Stage	Average R^2^ of the Self-Induction Coil
G1	G1-1	0.9081	0.9030	0.9215
	G1-2	0.9261	0.9329	
	G1-3	0.9165	0.9264	
	G1-4	0.9274	0.9405	
	G1-5	0.9163	0.9179	
G2	G2-1	0.9128	0.8629	0.9129
	G2-2	0.9249	0.9027	
	G2-3	0.9217	0.9113	
	G2-4	0.9273	0.9195	
	G2-5	0.9219	0.9240	
G3	G3-1	0.9307	0.9694	0.9341
	G3-2	0.9354	0.9244	
	G3-3	0.9450	0.9324	
	G3-4	0.9399	0.9205	
	G3-5	0.9412	0.9020	
